# Diabetes and Hypertension Differentially Affect Renal Catecholamines and Renal Reactive Oxygen Species

**DOI:** 10.3389/fphys.2019.00309

**Published:** 2019-04-16

**Authors:** Anna M. D. Watson, Eleanor A. M. Gould, Sally A. Penfold, Gavin W. Lambert, Putra Riza Pratama, Aozhi Dai, Stephen P. Gray, Geoffrey A. Head, Karin A. Jandeleit-Dahm

**Affiliations:** ^1^Department of Diabetes, Central Clinical School, Faculty of Medicine, Nursing and Health Sciences, Monash University, Melbourne, VIC, Australia; ^2^Baker Heart and Diabetes Institute, Melbourne, VIC, Australia; ^3^Iverson Health Innovation Research Institute, Faculty of Health, Arts and Design, Swinburne University of Technology, Hawthorn, VIC, Australia

**Keywords:** hypertension, diabetes, nephropathy, animal model, oxidative stress, catecholamines, monoamine oxidase

## Abstract

Patients with diabetic hypertensive nephropathy have accelerated disease progression. Diabetes and hypertension have both been associated with changes in renal catecholamines and reactive oxygen species. With a specific focus on renal catecholamines and oxidative stress we examined a combined model of hypertension and diabetes using normotensive BPN/3J and hypertensive BPH/2J Schlager mice. Induction of diabetes (5 × 55 mg/kg streptozotocin i.p.) did not change the hypertensive status of BPH/2J mice (telemetric 24 h avg. MAP, non-diabetic 131 ± 2 vs. diabetic 129 ± 1 mmHg, n.s at 9 weeks of study). Diabetes-associated albuminuria was higher in BPH/2J vs. diabetic BPN/3J (1205 + 196/-169 versus 496 + 67/-59 μg/24 h, *p* = 0.008). HPLC measurement of renal cortical norepinephrine and dopamine showed significantly greater levels in hypertensive mice whilst diabetes was associated with significantly lower catecholamine levels. Diabetic BPH/2J also had greater renal catecholamine levels than diabetic BPN/3J (diabetic: norepinephrine BPN/3J 40 ± 4, BPH/2J 91 ± 5, *p* = 0.010; dopamine: BPN/3J 2 ± 1; BPH/2J 3 ± 1 ng/mg total protein, *p* < 0.001 after 10 weeks of study). Diabetic BPH/2J showed greater cortical tubular immunostaining for monoamine oxidase A and cortical mitochondrial hydrogen peroxide formation was greater in both diabetic and non-diabetic BPH/2J. While cytosolic catalase activity was greater in non-diabetic BPH/2J it was significantly lower in diabetic BPH/2J (cytosolic: BPH/2J 127 ± 12 vs. 63 ± 6 nmol/min/ml, *p* < 0.001). We conclude that greater levels of renal norepinephrine and dopamine associated with hypertension, together with diabetes-associated compromised anti-oxidant systems, contribute to increased renal oxidative stress in diabetes and hypertension. Elevations in renal cortical catecholamines and reactive oxygen species have important therapeutic implications for hypertensive diabetic patients.

## Introduction

Diabetic patients often have concomitant conditions such as hypertension with such patients having a greatly accelerated development of nephropathy (Bakris et al., 2000). With the prevalence of diabetes world-wide predicted to increase to 642 million by 2040 (Ogurtsova et al., 2017) an increase in this more severe form of diabetic nephropathy is also likely to occur.

Renal catecholamines are critical contributors to the control of fluid and electrolyte homeostasis, particularly via renal proximal tubular adrenergic α- and β-receptors (Feraille and Doucet, 2001). The neural catecholamine, norepinephrine, modulates glomerular filtration rate directly, as well as influencing release of humoral effectors including renin and prostaglandin. Renal sympathetic nerves, via noradrenaline can directly increase renal tubular sodium reabsorption (Berne et al., 1952;Johns et al., 2011). Furthermore, noradrenaline induces increases of sodium-glucose cotransporter 2 and the glucose transporter GLUT2 gene expression in HK2 cells *in vitro* (Rafiq et al., 2015) while renal denervation decreased protein levels of GLUT2 in diabetic and non-diabetic rat kidney cortex (Schaan et al., 2005). In the kidney the catecholamine dopamine is predominantly produced by renal tubules where it too stimulates renin release and can cause natriuresis, as well as inhibiting tubuloglomerular feedback (for review see Zhang et al., 2009; Armando et al., 2011).

Renal sympathetic nerves also play a key role in hypertension however what occurs in diabetes with concomitant hypertension is less clear. Renal nerves are associated with the development of hypertension, both in humans (Esler et al., 1988) and in animal models such as the spontaneously hypertensive rat (SHR) (Kline et al., 1980). Increases in efferent renal sympathetic nerve activity have been associated with hypertension in rodents (Burgi et al., 2011) as well as renal norepinephrine content (Donohue et al., 1988) being significantly greater in hypertensive rats. Similarly, the interaction between dopaminergic D1- and D2 like receptors, the renin-angiotensin system, and renal sodium reabsorption, have long been linked to hypertension (Carey, 2013; Zhang and Harris, 2015).

Fluctuations in blood glucose levels can lead to compromised sympathetic and motor neuronal function and neural death. Peripheral diabetic neuropathy has also been linked to amputation rates in diabetic patients (Obrosova, 2009). Additionally, diabetes-induced elevations in reactive oxygen species and attenuation of antioxidant systems result in peripheral neuropathy (Obrosova, 2009). Renal sympathetic nerve activity is known to be lower in a rodent model of type 1 diabetes (Gu et al., 2009). Renal denervation of diabetic rats in early diabetes (2 weeks) attenuates the expansion of glomerular volume as well as reducing glomerular hyperfiltration (Luippold et al., 2004). Alternatively severing of the renal sympathetic nerves in streptozotocin-induced diabetic rodents after 6 weeks of diabetes led to an increase in albumin excretion (Matsuoka, 1993), indicating that renal nerves may be protective in diabetic nephropathy. Despite these studies, joint investigation of intrarenal norepinephrine and dopamine has, to our knowledge, never been reported in concomitant diabetes and hypertension.

In the 1970s genetically hypertensive mice (BPH/2J) and its normotensive sister-strain (BPN/3J) were created by selection of a hypertensive phenotype after inbreeding 8 different background strains (Schlager, 1974). More recently we have found that levels for the neural marker, tyrosine hydroxylase (TH), were significantly elevated in the kidney of hypertensive Schlager mice when compared to their normotensive counterparts (Jackson et al., 2013). We hypothesize that increases in renal catecholamines contribute to the development of hypertensive-diabetic nephropathy. Thus, in the current study, we aimed to assess the effects of diabetes on renal structure, renal function, renal catecholamines and oxidative stress in streptozotocin-induced diabetic Schlager mice, with and without concomitant hypertension.

## Materials and Methods

### Animals

Male hypertensive (BPH/2J) and normotensive (BPN/3J) Schlager mice were bred and housed at the Alfred Medical Research Education Precinct (AMREP) Animal Services animal house (Melbourne, VIC, Australia) under the approval of the AMREP ethics committee and with studies conducted according to Australian National Health and Medical Research Council guidelines in line with international standards.

All mice had unrestricted access to water and feed, were maintained on a 12 h light–12 h dark cycle and were fed standard mouse chow (Barastoc irradiated mice cubes, Ridley Corp., Melbourne, VIC, Australia) with water ad. Lib. Animals were randomly allocated to remain non-diabetic (control) or be rendered diabetic at 8 weeks of age by 5 daily i.p. injections of streptozotocin, 55 mg/kg (Sapphire BioScience; AdipoGen Life Sciences, Liestal, Switzerland) in citrate buffer. Animals were excluded from the study if weekly blood glucose levels (glycometer) were < 15 mmol/l and HbA1c < 53 mmol/mol (7%). Urine was collected (24 h metabolic caging) at 9 weeks of study and tissue and blood collected after 10 weeks of study (18 weeks old) in daylight hours.

### Telemetry BP

A subset of mice from each group had telemetric BP probes (model TA11PA-C10 Data Sciences International, St. Paul, MN, United States) surgically implanted with tips inserted into the aorta via the carotid artery as described previously (Butz and Davisson, 2001; Davern et al., 2009). Briefly, animals were inducted with 2–3% isoflurane and maintained at 0.5–1.5% isoflurane. Prior to surgery bupivacaine (1 mg/kg sub-dermal, Marcain^®^, Aspen Pharmacare, St. Leonards, NSW, Australia) was given at site of injection, betadine sprayed on the surgical site and carprofen (5 mg/kg s.c., Rimadyl^®^, Zoetis Ptd Ltd., Rhodes, NSW, Australia) given for 2 days post-op. along with fluid replacement (Hartmann’s solution (Baxter Healthcare Pty Ltd., Toongabbie, NSW, Australia) based on initial body weight). Animals were monitored for 10 days prior to recording. Twenty four hour BP were recorded 10 days after implantation at 9–10 weeks of study. These animals did not undergo metabolic caging and were transcardially perfused (see below for more detail; Supplementary Figure 1 for timeline).

### Animal End Point

After 10 weeks of study (18 weeks of age), in the light phase animals were weighed and anesthetized by an i.p. injection of sodium pentobarbitone (100 mg/kg body weight; Euthatal, Delvet Limited, Seven Hills, NSW, Australia). Blood was collected from the left ventricle, centrifuged and plasma and remaining blood cells were stored at -20°C and at 4°C, respectively. Erythrocytes were analyzed for glycated hemoglobin levels (Cobas b101, Roche Diagnostics, Mannheim, Germany). Plasma was assessed for lipid and glucose levels (Beckman Coulter LX20PRO Analyzer, Beckman Coulter Diagnostics, Mount Waverley, VIC, Australia) (Watson et al., 2012). Organs were rapidly dissected and weighed (wet weight) and kidneys were either snap frozen in liquid nitrogen and stored at -80°C, or stored in buffered formalin (10%, vol./vol.) before being embedded in paraffin. With the exception of BP, experiments were conducted in a blinded fashion. A timeline for procedures and measurements is provided (Supplementary Figure 1).

### RT-PCR

Snap frozen renal cortex was homogenized in trizol using 1 and 2 mm zirconium oxide beads and (Bullet Blender, Next Advance Inc., Troy, NY, United States). cDNA was prepared as described previously (Tikellis et al., 2003) and assessed for gene expression was by Taqman or fast SYBR Green [Applied Bio Systems (ABI), Prism 7500, Perkin-Elmer, Foster City, CA, United States, or a QuantStudio(TM) 7 Flex System, Thermo Fisher Scientific, Rockford, IL, United States]. Data was corrected for expression of 18S (ABI, Thermo Fisher Scientific) and expressed as fold expression relative to control normotensive animals. Sequences for the primers and probes used are listed in Supplementary Table 2.

### Functional Assays

Albuminuria was assessed by ELISA according to the manufacturer’s instructions (Bethyl Laboratories, Inc., Montgomery, TX, United States) (Watson et al., 2012).

Cystatin C (BioVendor – Laboratorni medicina a.s., Karasek, Brno, Czech Republic) and KIM-1 (kidney injury molecule-1, or hepatitis A virus cellular receptor 1; Assay Matrix; EIAab, Optics Valley, Wuhan, China) ELISA analyses were carried out on plasma samples as per the manufacturer’s instructions.

### Assessment of ROS

#### Dihydroethidium (DHE)

At 10 weeks of study animals which had undergone telemetric blood pressure measurement were injected with sodium pentobarbitone (as above) and transcardially perfused with 0.9% saline and ice-cold 4% paraformaldehyde/PBS (4% PFA) and transferred to 20% sucrose/TBS at 4°C overnight. Kidneys were frozen in OCT and stored at -20°C before sections being cut on a cryostat (20 μm).

As performed previously (Jha et al., 2017), frozen sections were thawed and further fixed for 15 min (4% PFA) before incubated in 10 μM DHE (Life Technologies, Eugene, OR, United States) (dissolved in DMSO and diluted in Krebs buffer) whilst being gently agitated for 40 min at 37°C. All sections were washed in phosphate buffered saline, rinsed with water and mounted (ProLong Gold anti-fade, Molecular Probes, Thermo Fisher Scientific). Images were captured immediately [Leica Eclipse Ni-U fluorescence microscope (LED), Nikon DS-Ri2 camera] and fluorescent brightness intensity quantitated (ImageJ v.1.52e, NIH, United States) and expressed relative to control normotensive animals.

#### Hydrogen Peroxide and Catalase Activity

Sample fractionation of renal cortical homogenate into mitochondrial and cytosolic fractions by centrifugation, as described previously (Coughlan et al., 2007). Hydrogen peroxide production (Amplex Red) (Life Technologies Australia/Thermo Fisher Scientific) and catalase activity (Abacus ALS; Cayman Chemical, Ann Arbar, MI, United States) were carried out on fractionated samples according to the manufacturer’s instructions.

### Western Blot

Western blot was run as performed previously (Jha et al., 2017). Reduced samples of kidney cortex (40 μg) were loaded onto a 4–20% acrylamide gel (Bio-Rad, Gladesville NSW, Australia) and incubated with primary antibody (MAO-A; monoclonal rabbit, Abcam Inc., Cambridge, MA, United States) and ECL (Sigma-Aldrich, St. Louis, MO, United States). Quantitation of blots was calculated relative to β-actin (monoclonal rabbit, Cell Signaling Technology, Genesearch, Arundel, QLD, Australia), using ImageLab (v.5.2.1, Bio-Rad) on a ChemiDoc Touch (Bio-Rad). See Supplementary Figure 5 for original blots.

### Staining and Analysis

Tubulointerstitial area (TIA) and mesangial expansion were calculated from sections stained with periodic acid-Schiff (PAS) as described previously (Calkin et al., 2006; Watson et al., 2012) using 13–17 images per section.

Sections (4 μm) of 10% formalin-fixed paraffin embedded kidney were used to immunohistochemically stain for TH (polyclonal rabbit, ab152, Merck-Millipore, Temecula, CA, United States) (Jackson et al., 2013), dopamine β hydroxylase (DBH; polyclonal rabbit, ab63939, Abcam), or monoamine oxidase A (MAO-A; monoclonal rabbit, Abcam). Dewaxed sections were incubated with 10% horse serum (NHS; Vector Laboratories, Burlingame, CA, United States) before being incubated overnight with primary antibody (in 1% NHS, 4°C). At room temperature sections were alternatively washed (tris buffered saline) and incubated with the appropriate biotinylated secondary antibody (Abacus ALS; Vector Laboratories), avidin-biotin complex (Vectastain Elite ABC kit, Abacus ALS; Vector Laboratories), and visualized with 3,3 Diaminobenzidine tetrahydrochloride (DAB; Sigma-Aldrich). Sections were then incubated at room temperature in periodic acid (5 min), washed in tap water, then Schiff reagent (5 min) and washed again. Sections were then dehydrated and mounted in DPX (Sigma-Aldrich).

Images (∼10/animal; Olympus BX-50, Olympus Optical; Q-imaging MicroPublisher 3.3 RTV camera, Surrey, BC, Canada) were taken under identical light conditions. The percentage area of staining (dark brown staining, assessed in RGB) in proximal or distal tubules were determined by circling cortical tubules where the brush boarder was either present or absent, respectively (Image Pro Analyzer, ver. 7.0, MediaCybernetics). PAS counterstaining did not affect DAB staining levels and negative controls were negative (see Supplementary Figure 3).

### Renal Catecholamine Content

Snap frozen cortex was homogenized in 4% perchloric acid (with 1% EDTA; both Sigma-Aldrich). Catecholaminergic content was assessed by HPLC as described previously (Lambert and Jonsdottir, 1998). Catecholaminergic content was corrected for total protein concentration of the final sample, as assessed by Pierce bicinchoninic acid (BCA) assay according to the manufacturer’s instructions (Thermo Fisher Scientific).

### Statistical Analysis

All data is expressed as mean ± SEM unless otherwise indicated. Statistical analysis was carried out by two-way ANOVA with *post hoc* with comparisons of group means performed by Tukey’s multiple comparison test with *p* < 0.05 considered significant (Prism v 7.02, GraphPad Software Inc., San Diego, CA, United States).

## Results

### Body Weight, Metabolic Caging, Plasma Lipids and Glucose

As expected, diabetes was associated with significantly lower body weight as well as increases in water intake and urine output (Table 1) while non-diabetic control hypertensive animals were lighter than normotensive mice. In both strains diabetic animals were similarly hyperglycemic. Diabetic mice with hypertension had a slight but significantly greater HbA1c level than diabetic normotensive animals [approximately +26 mmol/mol (∼+2%)], although at death, plasma glucose, lipids, water intake and urine output did not differ significantly between the two strains (*t*-test, *p* < 0.05). Overall two-way ANOVA results found strain to have a significant effect on total plasma cholesterol, water intake/body weight, and food consumption. After correction for body weight, *post hoc t*-tests found no significance differences between mouse strains when 24 h food intake, water intake or urine output was corrected for body weight.

**Table 1:** Physiological parameters for mice at the conclusion of the 10 week study (18 weeks of age) showing non-diabetic (control) normotensive mice are heavier than hypertensive animals.

Variable	Cont. Normot.	Cont. Hypert.	Diab. Normot.	Diab. Hypert.	*p*-value: Interaction	*p*-value: Effect of Diabetes	*p*-value: Difference Between Strains
Body Weight (g)	28 ± 0.6	23 ± 0.3^*^	24 ± 0.4^*^	22 ± 0.5	**<0.001**	**<0.001**	**<0.001**
HbA1c (mmol/mol)(%)	21 ± 1 (4.1 ± 0.1)	28 ± 1 (4.7 ± 0.1)	85 ± 5 (9.9 ± 0.4) ^∗^	111 ± 4 (12.3 ± 0.4) †‡	**<0.001**	**<0.001**	**<0.001**
Plasma glucose (mmol/L)	8.0 ± 0.8	7.7 ± 0.5	25.5 ± 0.9^*^	23.2 ± 1.1 †	0.263	**<0.001**	0.144
Total Cholesterol (mmol/L)	2.8 ± 0.2	2.4 ± 0.2	3.5 ± 0.2	3.1 ± 0.2	0.815	**0.001**	**0.049**
24 h Water intake (ml)	3.8 ± 0.5	5.2 ± 0.5	15.3 ± 1.4^*^	18.0 ± 2.0 †	0.537	**<0.001**	0.078
% 24 h Water intake/BW (ml/0.01 g)	13.6 ± 2.0	21.8 ± 2.0	67.7 ± 6.8^*^	83.8 ± 11.1 †	0.498	**<0.001**	**0.042**
24 h Urine output (ml)	0.4 ± 0.3+	0.3 ± 0.1	11.0 ± 1.1^*^	11.5 ± 1.0 †	0.093	**<0.001**	0.748
% 24 h Urine output/BW (ml/0.01 g)	1.67 ± 1.14	1.32 ± 0.23	48.4 ± 5.0^*^	53.7 ± 6.1 †	0.442	**<0.001**	0.501
24 h Food consumption (g)	2.2 ± 0.4	3.4 ± 0.3	4.9 ± 0.2^*^	5.3 ± 0.1 †	0.287	**<0.001**	**0.025**
24 h Food consumption/BW (g/g)	0.08 ± 0.02	0.14 ± 0.02	0.22 ± 0.01^*^	0.25 ± 0.01 †	0.294	**<0.001**	**0.005**
Heart weight (wet; mg)	152.2 ± 7.7	149.5 ± 10.0	132.7 ± 17.4	156.2 ± 16.2	0.301	0.619	0.282
Heart weight/BW (mg/g)	5.3 ± 0.2	6.3 ± 0.3	5.9 ± 0.8	7.5 ± 0.8	0.519	0.166	**0.024**
Left kidney weight (wet; mg)	234.8 ± 5.9	186.1 ± 4.9^*^	267.2 ± 4.7^*^	265.5 ± 12.8 †	**0.005**	**<0.001**	**0.001**
Left kidney weight/BW (mg/g)	8.2 ± 0.1	7.9 ± 0.1	11.8 ± 0.3^*^	12.6 ± 0.5 †	**0.036**	**<0.001**	0.146
Right kidney weight (wet;mg)	249.8 ± 8.6	197.1 ± 5.1^*^	290.9 ± 8.7^*^	269.6 ± 16.6 †	**0.015**	**<0.001**	**0.018**
Right kidney weight/BW (mg/g)	8.8 ± 0.2	8.3 ± 0.2	12.9 ± 0.6^*^	12.8 ± 0.2 †	0.427	**<0.001**	0.121


*As expected diabetic strains had elevations in plasma glucose and total cholesterol with no difference between strains, diabetic hypertensive animals had a slightly higher HbA1c. Data represents mean ± SEM. Body weight (BW) *n* = 21–29; organ weights *n* = 6–10, 24 h metabolic caging data *n* = 7–10; HbA1c *n* = 12–18, plasma cholesterol and glucose *n* = 13–17; plasma triglycerides 6–9. Cont., non-diabetic Control; Diab., diabetic; Normot., normotensive BPN/3J Schlager mice; Hypert., hypertensive BPH/2J Schlager mice. +, please note for urine volume only 4 Cont. Normot. animals produced urine. Significant *p*-values found with 2-factor ANOVA are in bold. *t*-test *p* < 0.05: ^∗^ vs. Cont. Normot.; † vs. Cont. Hypert.; ‡ vs. Diab. Normot.*

Wet kidney weights were significantly lower in non-diabetic (control) normotensive mice as compared to hypertensive animals but did not differ when corrected for body weight. Similar to previous studies with streptozotocin-induced diabetic mice (Watson et al., 2012), both diabetic strains displayed significant renal hypertrophy (associated with diabetes) (Table 1).

### Gene Expression

#### Fibrotic Markers

Overall two-way ANOVA results found significant differences between hypertensive and normotensive strains for *acta2* (α2 smooth muscle actin), *col4a1* (collagen IV), *ctgf, vegfa* (VEGF), and *rela* (p65 subunit of NFκB) (Table 2). Expression of *fn1* (fibronectin) was significantly greater in diabetic hypertensive mice compared to diabetic normotensive animals. Gene expression for *tgfb1, ednra, ednrb* (endothelin receptors) did not change significantly between groups at this time point (Supplementary Table 1).

**Table 2:** Gene expression for markers of fibrosis, components of the renin-angiotensin system, the catecholaminergic system and pro- and anti-oxidant systems in renal cortex of mice.

Gene	Cont. Normot.	Cont. Hypert.	Diab. Normot.	Diab. Hypert.	*p*-value: Interaction	*p*-value: Effect of diabetes	*p*-value: difference between strains
**Fibrotic**							
* acta2* (α2 smooth muscle actin)	1.0 ± 0.1	1.7 ± 0.3	1.3 ± 0.3	2.0 ± 0.4	0.921	0.208	**0.023**
*fn1* (fibronectin)	1.0 ± 0.1	0.9 ± 0.2	2.0 ± 0.4	2.4 ± 0.5 ‡	0.485	**0.002**	0.719
*col4a1* (collagen IV)	1.0 ± 0.2	1.6 ± 0.4	1.3 ± 0.4	2.1 ± 0.4	0.729	0.302	**0.043**
*ctgf*	1.0 ± 0.2	2.5 ± 0.7	1.2 ± 0.3	4.3 ± 1.5	0.429	0.334	**0.028**
*vegfa* (VEGF)	1.0 ± 0.2	1.5 ± 0.2	0.8 ± 0.2	1.4 ± 0.1	0.997	0.353	**0.004**
*rela* (p65 subunit of NFκB)	1.0 ± 0.2	2.4 ± 0.4	1.5 ± 0.5	3.4 ± 0.6	0.559	0.141	**0.003**
**Renin-Angiotensin System**							
*ren1* (renin)	1.0 ± 0.1	1.0 ± 0.3	1.1 ± 0.2	0.7 ± 0.1	0.311	0.690	0.365
*ace*	1.0 ± 0.1	1.6 ± 0.2^*^	0.5 ± 0.1	0.6 ± 0.1 †	0.187	**<0.001**	0.060
*agtr1*(AT1A receptor)	1.0 ± 0.2	1.7 ± 0.3	1.5 ± 0.4	1.0 ± 0.3	0.678	0.723	0.051
*agtr2* (AT2 receptor)	1.0 ± 0.2	3.8 ± 1.1^*^	1.5 ± 0.2	4.8 ± 0.7 ‡	0.718	0.234	**< 0.001**
**Catecholaminergic**							
*dbh*	1.0 ± 0.2	1.9 ± 0.3	1.4 ± 0.4	1.8 ± 0.5	0.507	0.778	0.088
*adra1b* (α adrenoceptor 1b subunit)	1.0 ± 0.1	1.3 ± 0.2	1.7 ± 0.2	2.4 ± 0.3	0.998	**0.013**	0.272
*adrb1* (β1 adrenoceptor)	1.0 ± 0.1	0.7 ± 0.1	1.3 ± 0.3	0.8 ± 0.1	0.383	0.281	**0.008**
*slc6a2* (norepinephrine transporter)	1.0 ± 0.1	0.7 ± 0.2	2.1 ± 0.3^*^	2.1 ± 0.3	0.229	**0.003**	0.171
*comt*	1.0 ± 0.1	1.2 ± 0.2	1.6 ± 0.4	1.5 ± 0.2	0.424	0.069	0.868
*maoa*	1.0 ± 0.2	1.0 ± 0.4	2.0 ± 0.4	3.8 ± 0.9 †	0.131	**0.003**	0.118
*maob*	1.0 ± 0.2	0.7 ± 0.1	1.4 ± 0.5	0.9 ± 0.1	0.624	0.191	0.054
**Pro-oxidant**							
*nox1*	1.0 ± 0.2	1.6 ± 0.4	3.1 ± 1.1	3.0 ± 0.8	0.561	**0.005**	0.675
*cybb* (Nox2)	1.0 ± 0.1	0.8 ± 0.2	1.4 ± 0.4	1.0 ± 0.2	0.674	0.152	0.165
*nox4*	1.0 ± 0.1	2.7 ± 0.8	0.9 ± 0.2	2.2 ± 0.4	0.691	0.548	**0.005**
*ncf1* (p47phox)	1.0 ± 0.1	0.7 ± 0.1	1.1 ± 0.2	1.3 ± 0.2 †	0.084	**0.010**	0.523
**Anti-oxidant**							
*sod1* (CuZnSOD; SOD1)	1.0 ± 0.1	1.1 ± 0.1	0.9 ± 0.2	1.1 ± 0.1	0.948	0.732	0.373
*sod2* (MnSOD; SOD2)	1.0 ± 0.2	1.0 ± 0.2	0.7 ± 0.1	1.2 ± 0.1	0.119	0.707	0.086
*cat* (catalase)	1.0 ± 0.1	1.5 ± 0.2^*^	0.9 ± 0.1	1.5 ± 0.1 ‡	0.797	0.507	**0.001**


*The protein encoded by the gene is in brackets. *n* = 3–9. Data presented as mean ± SEM. Significant *p*-values found with 2-factor ANOVA are in bold. *t*-test *p* ≤ 0.05: ^∗^ vs. Cont. Normot.; † vs. Cont. Hypert.; ‡ vs. Diab. Normot.; Note- *rela* Diab. Normot. vs. Diab. Hypert. *p* = 0.057, *nox4* Cont. Normot. vs. Cont. Hypert. *p* = 0.066.*

#### Renin-Angiotensin System

Two-way ANOVA found diabetic status to significantly affect gene expression for *ace* while *t*-tests found hypertensive mice to have significantly greater levels of both *ace* and *agtr2* (AT2; nearly fourfold higher) than normotensive mice irrespective of diabetic status, suggesting increases in renal gene expression of *agtr2* may be related to hypertensive status. Gene expression of *ace2*, and *mas1* did not differ significantly between groups (Supplementary Table 1).

#### Catecholaminergic System

*dbh* (encoding DBH, which catalyzes the production of norepinephrine from dopamine) expression did not differ significantly between groups (Table 2). Levels of *th* were undetectable in most samples, except those of diabetic hypertensive animals (data not shown). Two-way ANOVA showed diabetic status to have a significant effect on *adra1b* (α adrenoceptor 1b) with diabetic hypertensive animals having the greatest expression levels. Overall mouse strain had a significant effect on *adrb1* (β1 adrenoceptor) expression. Diabetic normotensive animals had greater *slc6a2* (norepinephrine transporter) expression compared to control normotensive animals. Expression for the catecholamine metabolizing enzymes *comt* and *maob* did not differ between these two groups. Expression for *maoa* was significantly greater in diabetic hypertensive animals when compared to non-diabetic hypertensive mice (nearly fourfold higher).

#### Oxidant and Anti-oxidant Markers

Two-way ANOVA analysis found that overall strain affected gene expression of *nox4* while diabetic status affected gene expression of *nox1* and *ncf1* (p47phox), with *t*-tests finding diabetic hypertensive mice had significantly greater *ncf1* renal cortical gene expression than control hypertensive animals (Table 2). *cybb* (nox2) expression was not significantly different between any groups. Hypertensive mice had significantly greater *cat* (catalase) expression than normotensive animals irrespective of diabetic status.

### Renal Function and Injury

**FIGURE 1:**
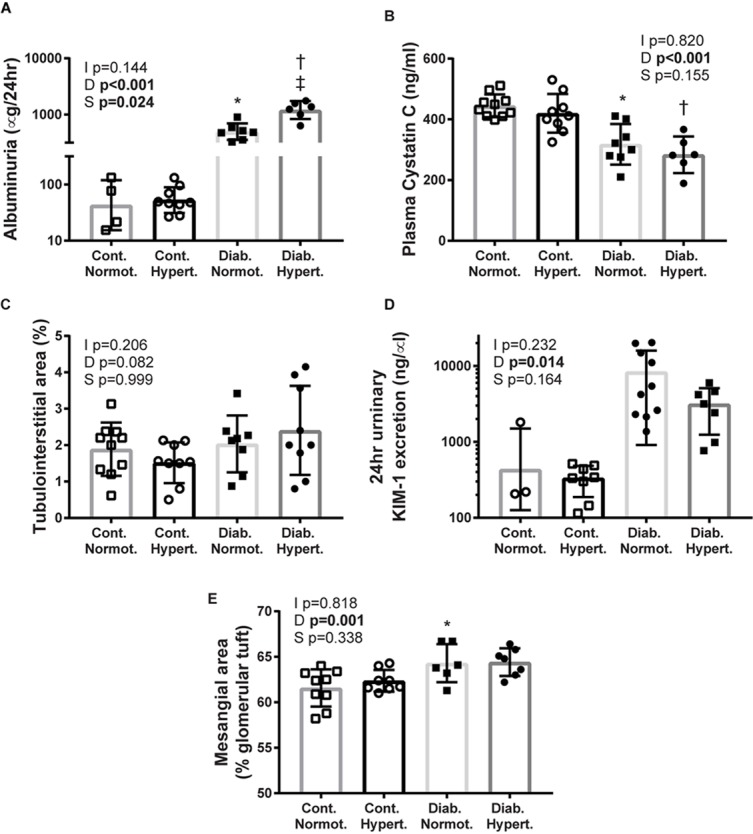
Markers of renal injury: albuminuria (**A**; geometric means ± geometric SD), plasma cystatin C **(B)**, tubulointerstitial area **(C)**, urinary kidney injury molecule-1 (KIM-1) **(D)**, mesangial expansion **(E)**. Diabetes resulted in significant differences for albuminuria, plasma cystatin C, KIM-1, and mesangial expansion. While hypertensive diabetic mice had much greater albuminuria there were no significant differences between the two diabetic groups for the other measures of renal injury assessed. Cont., non-diabetic control; Diab., diabetic; Normot., normotensive BPN/3J mice; Hypert., hypertensive BPH/2J mice. *n* = 5–10/group except for control normotensive animals: *n* = 3 urinary KIM-1, *n* = 4 albuminuria. 2-factor ANOVA *p*-values: I, interaction; D, relative to diabetic status; S, relative to strain. *T*-test *p* < 0.05: ^∗^ vs. Cont. Normot.; † vs. Cont. Hypert.; ‡ vs. Diab; Mesangial area Cont. Hypert. vs. Diab. Hypert. *p* = 0.07.

Albuminuria was significantly greater in both diabetic strains, however, diabetic hypertensive animals had ∼2–3 fold greater urinary albumin levels than diabetic normotensive mice (Figure 1A). It is not likely that this difference was due to greater glomerular filtration rate as both diabetic hypertensive and diabetic normotensive mice had similar levels of plasma cystatin C, this being significantly less than the respective non-diabetic control mice (Figure 1B), indicating glomerular hyperfiltration. After 10 weeks of diabetes hypertensive mice showed no significant difference in TIA compared to control hypertensive animals (Figure 1C). Two-way ANOVA analysis found that diabetic status significantly affected levels of the tubular injury marker KIM-1 although there was no significant difference between the two diabetic strains (Figure 1D). Thus, by these measures, tubular injury was not found to differ between diabetic hypertensive and diabetic hypertensive mice.

Diabetic status overall affected mesangial expansion with diabetic normotensive animals having significantly greater levels of mesangial expansion than their non-diabetic control normotensive mice (Figure 1E). The difference between diabetic and control hypertenive mice did not reach significance (*p* = 0.07). There was no difference in mesangial area between the two strains of diabetic animals indicating that concomitant diabetes and hypertension did not result in greater mesangial expansion at this timepoint.

### Blood Pressure

**FIGURE 2:**
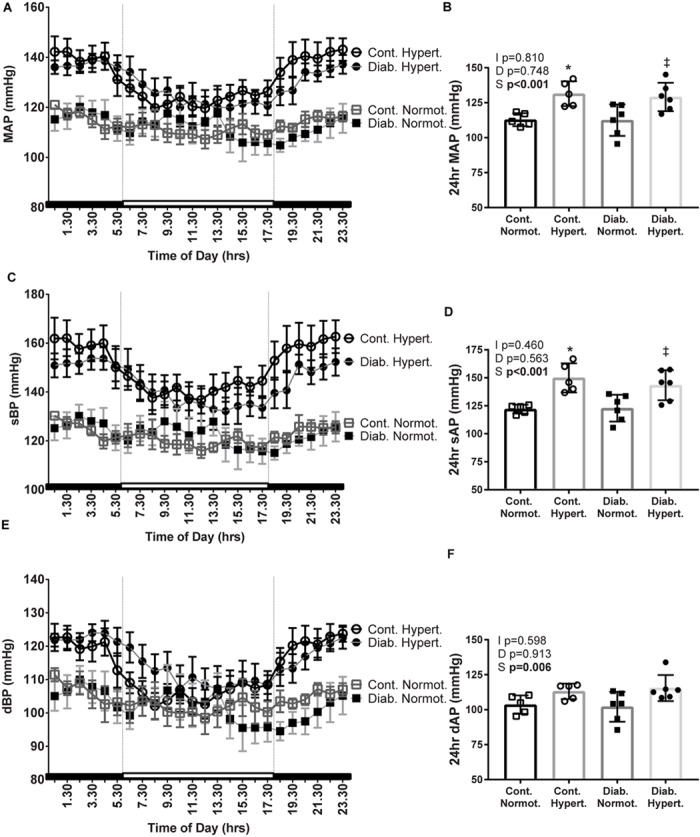
Mean arterial pressure (MAP; **A,B**), systolic BP (sBP; **C,D**), and diastolic BP (dBP; **E,F**), 24 h recordings were taken from conscious mice implanted with telemetric blood pressure probes after 10 weeks of study (24 h averages: **B,D,F**). Diabetic hypertensive mice maintained a high blood pressure despite polyuria and polydipsia. *n* = 3–9/group. Mean ± SEM. 2-factor ANOVA *p*-values: I, interaction; D, relative to diabetic status; S, relative to strain. *t*-test *p* < 0.05: ^∗^ vs Cont. Normot.; ‡ vs Diab. Normot.

Despite the fluid perturbations from diabetes associated polydipsia and polyuria (Table 1) mice were still hypertensive in both the light (inactive) and dark (active) phases, as well as over 24 h, with diabetic hypertensive animals having a significantly greater MAP, and sAP than diabetic normotensive mice (Figure 2).

### Renal Catecholamines

**FIGURE 3:**
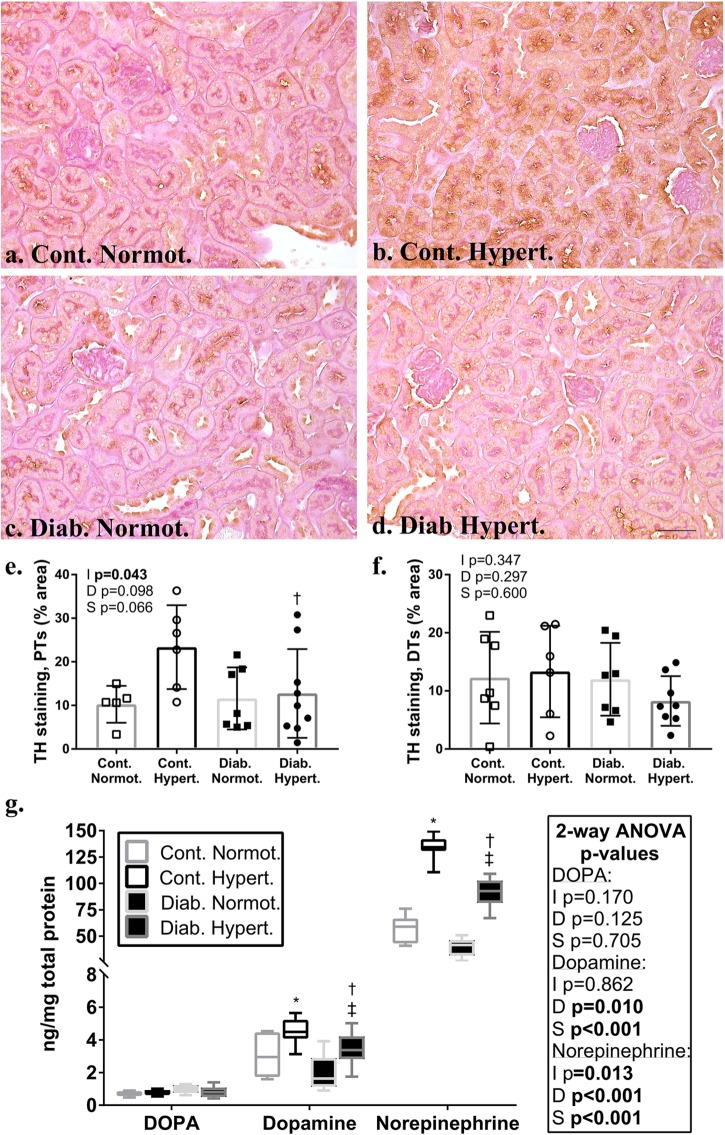
Immunohistochemical localization in the renal cortex of the rate limiting enzyme in catecholamine production, tyrosine hydroxylase (TH; **a–d;** brown stain) in non-diabetic control and diabetic normotensive and hypertensive mice. Periodic acid Schiff’s (PAS) stain (pink) was used as a counterstain to distinguish proximal (PTs) and distal tubule (DTs) populations. Quantification of proximal tubules **(e)** and distal tubules **(f)** shows non-diabetic control hypertensive mice had a greater percentage area of staining for TH in proximal tubules compared to diabetic hypertensive animals. There were no significant differences between groups for the percentage area stained for TH in distal tubules. Renal cortical catecholamine content **(g)** for DOPA, dopamine, and norepinephrine. Note change in scale for norepinephrine. Hypertensive animals had significantly greater amounts of norepinephrine and dopamine. Diabetic animals had significantly less dopamine and norepinephrine content. Diabetic hypertensive mice retained significantly greater levels of dopamine and norepinephrine as compared to diabetic normotensive mice. *n* = 6–9/group. 2-factor ANOVA *p*-values: I=interaction, D=relative to diabetic status, S=relative to strain. *t*-test *p* < 0.05: ^∗^ vs. Cont. Normot.; † vs. Cont. Hypert.; ‡ vs. Diab. Normot; TH staining in PTs Cont. Normot. vs. Cont. Hypert *p* = 0.06. DOPA- L-3,4-dihydroxyphenylalanine. Scale bar: 50 μm.

Immunohistochemical staining for the catecholaminergic rate limiting enzyme, TH, showed a trend toward significance (*t*-test, *p* = 0.061) in the proximal tubules of non-diabetic control hypertensive mice as compared to control normotensive animals (Figure 3a–f). Diabetic hypertensive animals (Figure 3e) had significantly less TH staining in proximal tubules as compared to control hypertensive mice with two-way ANOVA testing found a significant interaction between diabetic status and strain. There was no significant difference in staining levels of TH in the distal tubule between any of the groups (Figure 3f). Immunohistochemical staining for DBH showed no significant differences in distribution in proximal or distal tubules between any of the groups (Supplementary Figure 2). Counterstaining with PAS did not affect DAB staining levels and use of a non-immune IgG primary antibody resulted in negligible staining (Supplementary Figure 3).

Renal cortical levels, of both dopamine and norepinephrine, were higher in hypertensive compared to normotensive animals, irrespective of diabetic status (Figure 3g). Two-way ANOVA showed diabetic status had a significant effect on both dopamine and noradrenaline levels in the renal cortex with diabetic hypertensive mice having significantly less dopamine and norepinephrine than non-diabetic controls. Levels of both were still significantly greater in diabetic hypertensive animals as compared to diabetic normotensive animals. There was no significant difference in renal cortical L-3,4-dihydroxyphenylalanine (DOPA) levels between groups.

**FIGURE 4:**
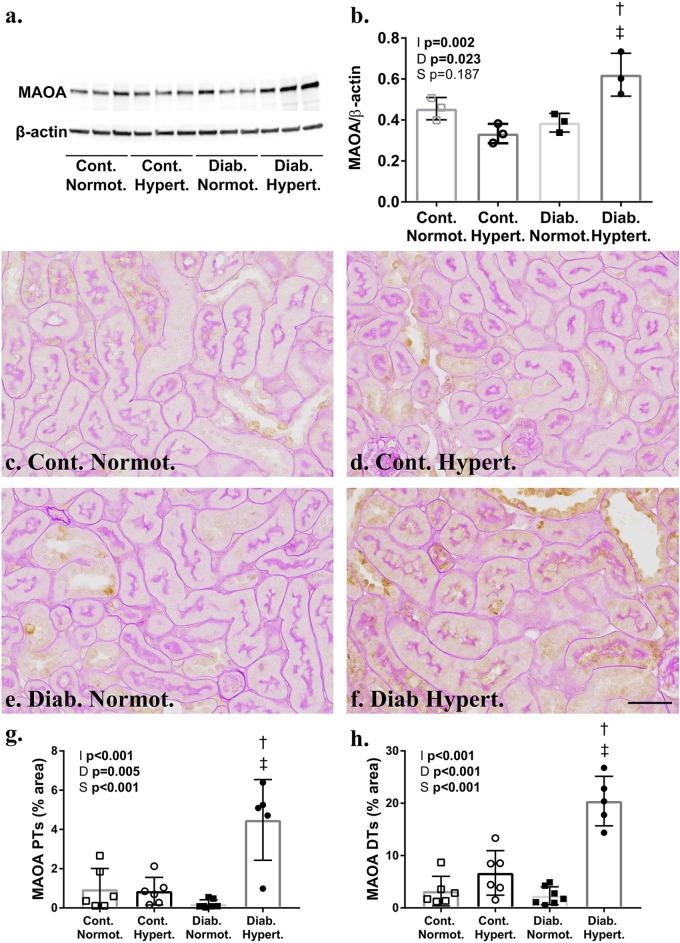
Western blot analysis **(a,b)** confirmed significantly greater levels of monoamine oxidase A (MAO-A) (60 kDa) in the renal cortex of diabetic hypertensive animals after correction for β-actin. Immunohistochemical staining for MAO-A **(c–f)** was significantly greater in the proximal (PTs; **g**) and distal tubules (DTs) of hypertensive mice **(h)**. **(b)** *n* = 3/group; **(g,h)** *n* = 7–8/group. 2-way ANOVA *p*-values: I, interaction; D, relative to diabetic status; S, relative to strain. *t*-test *p* < 0.05: † vs. Cont. Hypert.; ‡ vs. Diab. Normot. Cont-, non-diabetic control; Diab., diabetic; Normot., normotensive BPN/3J mice; Hypert., hypertensive BPH/2J mice. Scale bar: 100 μm. See Supplementary Data for whole blots.

Western blot analysis of whole renal cortex showed significant increases in the catecholaminergic metabolizing enzyme, MAO-A (Figure 4a,b). Immunohistochemical staining for MAO-A confirmed this with significantly greater staining in both proximal and distal tubules of diabetic hypertensive mice compared to both control hypertensive and diabetic normotensive animals (Figure 4g,h).

### Cortical Renal Oxidant and Anti-oxidant Systems

**FIGURE 5:**
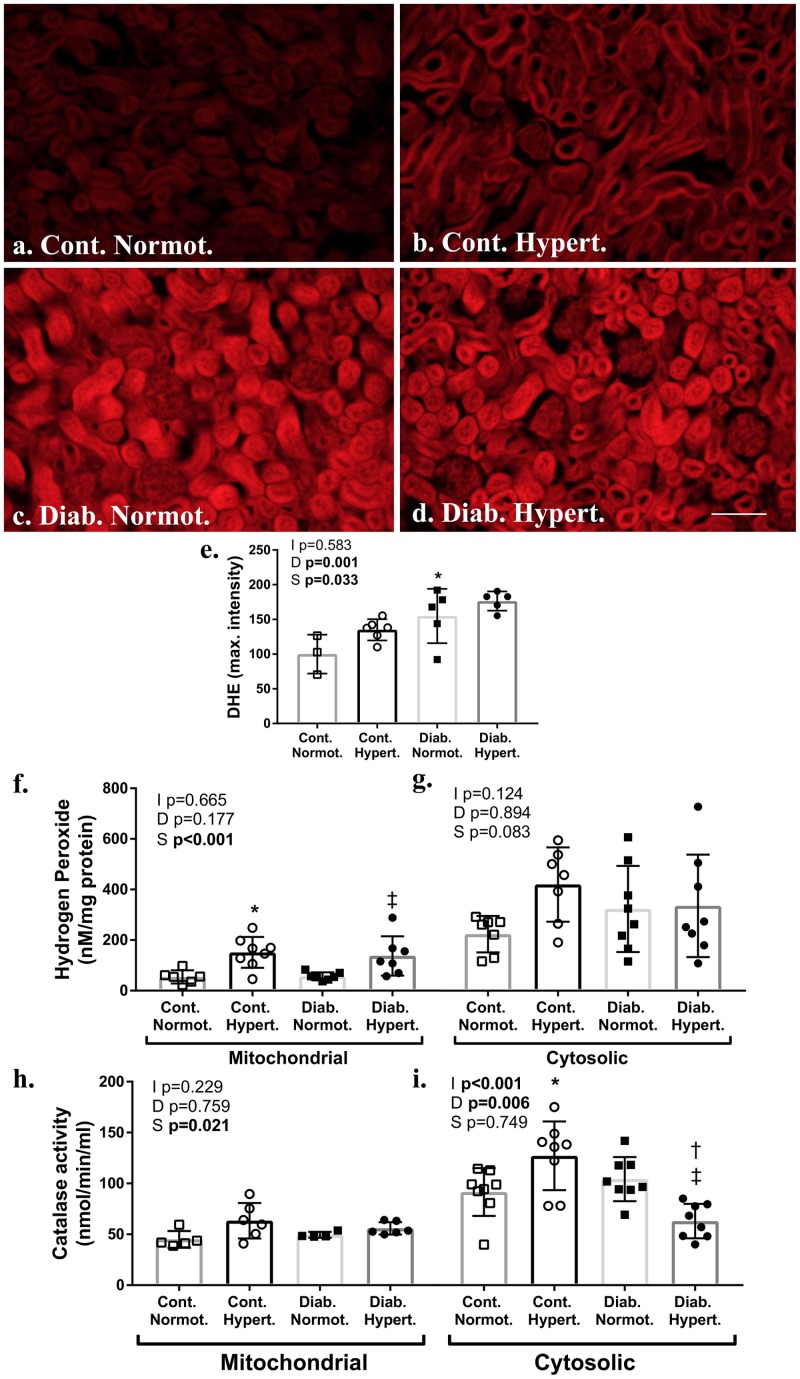
Measures of renal cortical oxidant and anti-oxidant systems. Assessment of frozen sections for superoxide by dihydroethidium (DHE; **a–e**) found significantly greater fluorescence intensity in diabetic animals relative to non-diabetic control hypertensive mice **(e)**. Assessment of renal cortical cytosolic and mitochondrial fractions for hydrogen peroxide production **(f,g)** found hypertensive animals had significantly greater mitochondrial levels irrespective of diabetic status. Catalase activity **(h,i)** was significantly greater in the cortical mitochondrial and cytosolic fraction of diabetic normotensive animals as compared to non-diabetic normotensive mice. The cytosolic fraction of diabetic hypertensive mice had significantly less catalase activity than diabetic normotensive mice. DHE *n* = 5–6/group except control normotensive *n* = 3; Hydrogen peroxide production and catalase activity *n* = 6-8/group. Normot. Cont- non-diabetic control; Diab., diabetic; Normot., normotensive BPN/3J mice; Hypert., hypertensive BPH/2J mice. 2-way ANOVA *p*-values: I, interaction; D, relative to diabetic status; S, relative to strain. *t*-test *p* < 0.05: ^∗^ vs. Cont. Normot.; † vs. Cont. Hypert.; ‡ vs. Diab. Normot.; DHE Cont. Hypert. vs. Diab. Hypert. *p* = 0.07. Scale bar: 100 μm.

Two-way ANOVA found a significant effect of both diabetic status and mouse strain for levels of DHE fluorescence in the renal cortex. Diabetic normotensive mice had significantly greater maximal fluorescence intensity compared to respective non-diabetic control animals (Figure 5a–e) indicating diabetes induces an increase in superoxide production in cortical renal tubules. *Post hoc t*-test found no significant difference in DHE intensity between diabetic strains.

Western blot showed levels of NADPH oxidase isoforms Nox 2 and Nox4 to be significantly greater in renal cortical samples from control hypertensive mice compared to control normotensive animals (Supplementary Figure 4). Nox4 levels were not significantly elevated in diabetic hypertensive mice, and in fact were significantly lower. Similarly, no significant elevations were found for other Nox isoforms with Nox1 and Nox2 also being lower with no change in p47phox (Supplementary Figure 4). This is not an assessment of enzyme activity levels however.

Hydrogen peroxide levels, produced by enzymes including Nox 4 and MAO-A, were significantly increased in mitochondrial fractions of the renal cortex from hypertensive mice, as compared to normotensive mice for both non-diabetic control and diabetic animals (Figure 5f). Activity for catalase, the enzyme that converts hydrogen peroxide into water and oxygen, was increased in both the cytosolic fraction of renal cortex from non-diabetic control hypertensive animals as compared to control normotensive mice (Figure 5g). Catalase activity (Figure 5h,i) was significantly lower in the cytosolic fraction of renal cortex from diabetic hypertensive mice as compared to non-diabetic hypertensive and diabetic normotensive mice with two-way ANOVA finding a significant interaction between diabetic status and strain. Two-way ANOVA found strain to have a significant effect on catalase activity in mitochondrial fractions however *post hoc t*-tests found no specific intergroup differences.

A summary comparing diabetic, hypertensive, and diabetic hypertensive animals is provided in Supplementary Table 3.

## Discussion

Diabetic patients with concomitant hypertension have an accelerated rate of nephropathy (Bakris et al., 2000). While renal nerves and renal catecholamines are known to be altered in hypertension, the effects of diabetes upon these renal control systems are less well studied. To the best of our knowledge this is the first study to compare renal dopamine and norepinephrine in diabetes with and without concomitant hypertension. We found that insulin-deficient diabetes was associated with significantly lower levels of renal dopamine and norepinephrine while hypertension was associated with significantly greater levels of these catecholamines. Although diabetic hypertensive animals maintained greater levels of both dopamine and norepinephrine than normotensive mice diabetic status was associated with lower levels of these catecholamines. This, together with the finding that diabetic hypertensive animals have elevations in reactive oxygen species whilst simultaneously having compromised antioxidant capacity (catalase activity), suggests that this combination may be an underlying cause for the significantly greater levels of albuminuria seen in these animals.

Both norepinephrine and dopamine play important roles in kidney function (Armando et al., 2011; Johns et al., 2011) as well as pathophysiological roles in hypertension (Esler et al., 1989; Armando et al., 2015; Grassi and Ram, 2016). Despite having similar effects in the kidney, renal dopamine and norepinephrine are seldom considered in conjunction. TH is the rate limiting enzyme in neuronal dopaminergic and noradrenergic production, converting tyrosine to L-DOPA (Eisenhofer et al., 2004). In the kidney TH is linked solely to noradrenergic innervation however as most renal dopamine is produced by renal tubules by direct uptake of L-DOPA into the tubule, not via TH mediated production (Armando et al., 2011). Previous autoradiographical studies with tritiated norepinephrine have shown extensive innervation of the basement membranes, with proximal tubules showing the greatest concentration of norepinephrine of all tubule populations (Barajas et al., 1984). SHR show greater TH immunostaining density in the renal arterioles of compared to normotensive Wistar-Kyoto controls as well as greater levels of renal norepinephrine (Burgi et al., 2011).

Previously we reported that immunostaining for TH was greater in the renal cortical tubular area of hypertensive Schlager mice (Jackson et al., 2013). Proximal tubules are responsible for most glucose and sodium reuptake (Feraille and Doucet, 2001) which correlates with alterations in neural control of fluid and solute uptake. Elevations in renal norepinephrine have also been associated with renal injury. In a high renal perfusion pressure model of renal injury, infusion of norepinephrine led to significantly greater levels of glomerular injury than angiotensin infusion despite animals having equivalent BPs (Polichnowski and Cowley, 2009).

It is important to note that diabetic hypertensive mice did not have elevations in proximal tubular TH, but rather levels were significantly lower than that seen in non-diabetic hypertensive animals. Levels of norepinephrine were still significantly higher in diabetic hypertensive mice than diabetic normotensive animals. Interestingly, our data show that renal dopamine levels show similar trends when compared to norepinephrine with respect to diabetes and hypertension. Dopamine production has been found to be lower in type-1 diabetic rats (Armando et al., 2011) and lower urinary excretion of both dopamine and norepinephrine has been reported in type 2 diabetic patients, with diabetic patients with nephropathy showing the lowest levels of urinary catecholamines (Murabayashi et al., 1989). Lower levels of norepinephrine and dopamine associated with diabetes may contribute to further pathological changes in the kidney in the long term.

**FIGURE 6:**
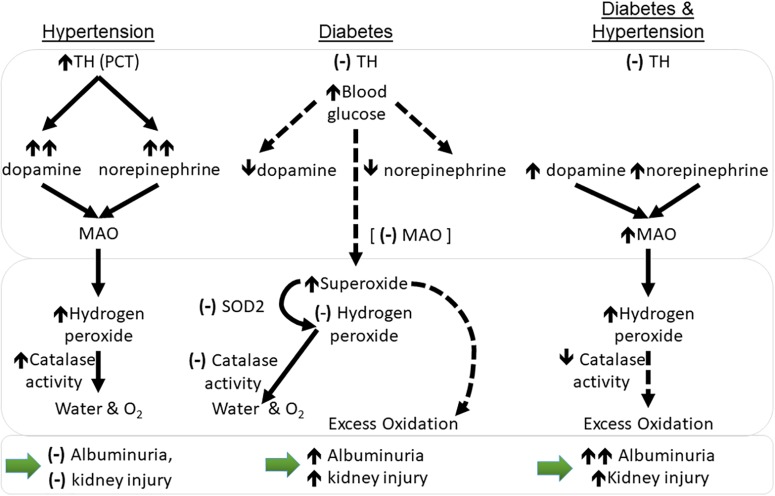
Working hypothesis relating to changes in catecholamines and oxidation in the kidney cortex of mice with hypertension and diabetes with respect to monoamine oxidase (MAO-A). Dotted lines represent theorized outcomes. (–): no change; ↑: increase in levels; ↑↑: large increase in levels; ↓: decrease in levels. PCT, proximal convoluted tubules; MAO, monoamine oxidase levels; *sod2*- superoxide dismutase 2 (MnSOD) gene transcript levels; TH, tyrosine hydroxylase protein levels. Large bottom arrows: relation to albuminuria and the overall level of kidney injury seen.

In the rat, MAO has been localized to renal proximal tubules and the collecting ducts, specifically the outer mitochondrial membranes, rough endoplasmic reticulum, and nuclear envelope (Muller and Da Lage, 1977) whilst renal distal tubules have shown moderate activity and low levels of staining for MAO in healthy guinea pig and humans, respectively (Glenner et al., 1957; Hodorova et al., 2012). Clinically, MAO inhibition is used to treat Parkinson’s disease anxiety and depression (although medications targeting MAO-B are preferred) (Edmondson et al., 2009; Thomas et al., 2015). In hypertensive mice, higher renal levels of both norepinephrine and dopamine likely lead to greater metabolism by MAO, although levels were not elevated. Diabetic hypertensive animals did have significantly greater renal tubular MAO-A compared to diabetic normotensive mice, however (Figure 4, 6). *In vitro* MAO-A activity has been associated with pro-apoptotic cascades via its production of hydrogen peroxide (Bianchi et al., 2003). We found hypertensive animals to have significantly greater levels of renal mitochondrial hydrogen peroxide production compared to normotensive mice, that is hydrogen peroxide production driven by hypertension, not diabetes *per se* (at least at this point in disease progression; at 10 weeks of study).

Diabetic mice have elevations in renal reactive oxygen species, including DHE (Jha et al., 2017). This is associated with elevations in the mitochondrial associated enzyme (NADPH oxidase) Nox4, an important contributor to the development of diabetic nephropathy (Jha et al., 2014), also produces hydrogen peroxide. We found that non-diabetic hypertensive animals also showed an increased gene and protein expression for Nox4 thus this may also be contributing to the observed greater levels of hydrogen peroxide.

Non-diabetic hypertensive animals also had an higher activity of the hydrogen peroxide metabolizing enzyme, catalase, which converts hydrogen peroxide into water and oxygen (O_2_). Immortalized proximal tubular cells from SHR have been found to have greater hydrogen peroxide production compared to normotensive control cells (Simao et al., 2008) although in this case it was linked to lower levels of catalase activity (Gomes et al., 2013). This did not occur in the current study. Diabetes results in compromised antioxidant systems and the present data reflects this; unlike non-diabetic controls, diabetic hypertensive animals did not show greater catalase activity. Indeed, when compared to diabetic normotensive animals, catalase activity was significantly lower in renal cytosolic fractions of diabetic hypertensive mice. Thus, any excess hydrogen peroxide production could then contribute to a greater accumulation of reactive intermediates and lead to greater renal damage, especially in the long term (Figure 6).

Whilst both normotensive and hypertensive Schlager mice are a well characterized model, this is the first study to report the effects of streptozotocin-induced diabetes in this model. In original characterization studies (1979) 14–21 week old hypertensive mice showed significantly greater heart and kidney weights (with and without correction for body weight) as compared to normotensive animals (Schlager et al., 1979). This was not the case in the present study (Table 1) where animals are 18 weeks old. Importantly both strains of mice in the original characterization study weighed more than the current study. Genetic drift could be expected between this initial study and the present time, however, it is interesting to note that in this case kidney size is not necessarily linked to hypertensive status. Older studies comparing non-diabetic Schlager mouse strains also found that by 10–20 weeks of age hypertensive mice have less body fluid (as a percentage of body weight) than normotensive Schlager mice (Rosenberg et al., 1985). In the present study we found no significant differences between strains (with and without concomitant diabetes) for water intake or urine output at 18 weeks of age (Table 1).

In the present study two-way ANOVA found strain to have a significant effect with greater food consumption. Hypertensive mice have previously been reported to be hyperactive compared to normotensive mice (Davern et al., 2009), thus energy expenditure may account for the difference in body weights. Similar to previous studies, diabetic mice had smaller body weights, greater water intake and urine output and renal hypertrophy when compared to their non-diabetic counterparts, as well as albuminuria and mesangial expansion (Watson et al., 2012). Hypertension alone did not result in greater mesangial expansion.

At this time point (10 weeks of study)/age (18 weeks old), non-diabetic (control) hypertensive mice did not show greater albuminuria than non-diabetic normotensive animals. Original characterization studies of hypertensive Schlager mice found that older hypertensive animals (43–50 weeks old) had a significantly smaller basement membrane mean width with numerous sub-epithelial focal thickenings compared to normotensive mice. There was no difference in epithelial slit pore width, however, (Rosenberg et al., 1979). This suggests that even with a longer hypertensive duration nephropathy is not severe in this model.

Both groups of diabetic mice developed albuminuria with diabetic hypertensive animals showing ∼3 fold greater albuminuria than diabetic normotensive mice. This may be partially accounted for by the slight but significantly greater HbA1c in the hypertensive strain, nevertheless fluid intake and output did not differ between strains nor did plasma glucose levels at death. Greater mechanical force at the damaged slit diaphragm could be expected to increase albumin excretion into the urine. Additionally, diabetic SHR show exaggerated albuminuria when compared to normotensive diabetic rats which was linked to exaggerated loss of glomerular nephrin, indicating greater podocyte loss in these animals (Forbes et al., 2002). Thus putative podocyte loss within the glomerulus may also contribute to the potentiation of albuminuria. The greater albuminuria in diabetic hypertensive mice was not accounted for by glomerular filtration, glomerular damage associated with mesangial expansion, or tubular damage as these measures were equivalent between the two diabetic strains, with and without concomitant hypertension.

## Conclusion

In this study we describe specific differences between diabetic mice with and without concomitant hypertension. In particular we demonstrate renal cortical levels of dopamine and norepinephrine were greatest in non-diabetic hypertensive mice whilst diabetes was associated with significantly lower levels of both these catecholamines. Diabetic mice with concomitant hypertension retain elevations in renal dopamine and norepinephrine levels although to a significantly reduced extent compared to non-diabetic hypertensive animals. Hypertension *per se* was not associated with albuminuria or renal damage at this time point however diabetic hypertensive mice displayed overt albuminuria. These data suggest diabetic mice with concomitant hypertension are particularly prone to oxidative stress in part due to increased catecholamine metabolism by MAO-A as well as reduced anti-oxidant capacity. Neither the renal catecholaminergic system, nor renal oxidative stress are current clinical priorities in patients with diabetes and concomitant hypertension. These findings suggest that oxidant and antioxidant systems should be approached as potential clinical targets, particularly in patients with concomitant diabetes and hypertension.

## Author Contributions

AW designed and performed the experiments, analyzed and collated the data, and wrote the manuscript. AW is responsible and accountable for all data presented, including the integrity of the data and the accuracy of the data analysis. KJ-D interpreted the data and wrote the manuscript. GH and GL interpreted the data and edited the manuscript. SP, EG, SG, AD, and PP performed the experiments, interpreted the data, and edited the manuscript. All authors provided approval for the publication of the content included.

## Conflict of Interest Statement

KJ-D has received research funding from Boehringer Ingelheim and GlaxoSmithKline. GL has acted as a consultant for Medtronic and has received honoraria or travel support for presentations from Pfizer, Wyeth Pharmaceuticals, Servier, and Medtronic. The remaining authors declare that the research was conducted in the absence of any commercial or financial relationships that could be construed as a potential conflict of interest.
