# Supplementary material for: Diabetes and Hypertension Differentially Affect Renal Catecholamines and Renal Reactive Oxygen Species
Source: Front Physiol. 2019 Apr 16;10:309. doi: 10.3389/fphys.2019.00309 (PMC6477025; doi:10.3389/fphys.2019.00309)
Supplement: Supplementary file 1 [file Table_1.DOCX]

Supplementary Material

Diabetes and Hypertension Differentially Affect Renal Catecholamines and Renal Reactive Oxygen Species

**Anna M.D. Watson*, Eleanor Gould, Sally A. Penfold, Gavin W. Lambert, Putra Riza Pramata, Stephen P. Gray, Geoffrey A. Head, and Karin A. Jandeleit-Dahm**

*** Correspondence:** [anna.watson@monash.edu](mailto:anna.watson@monash.edu)


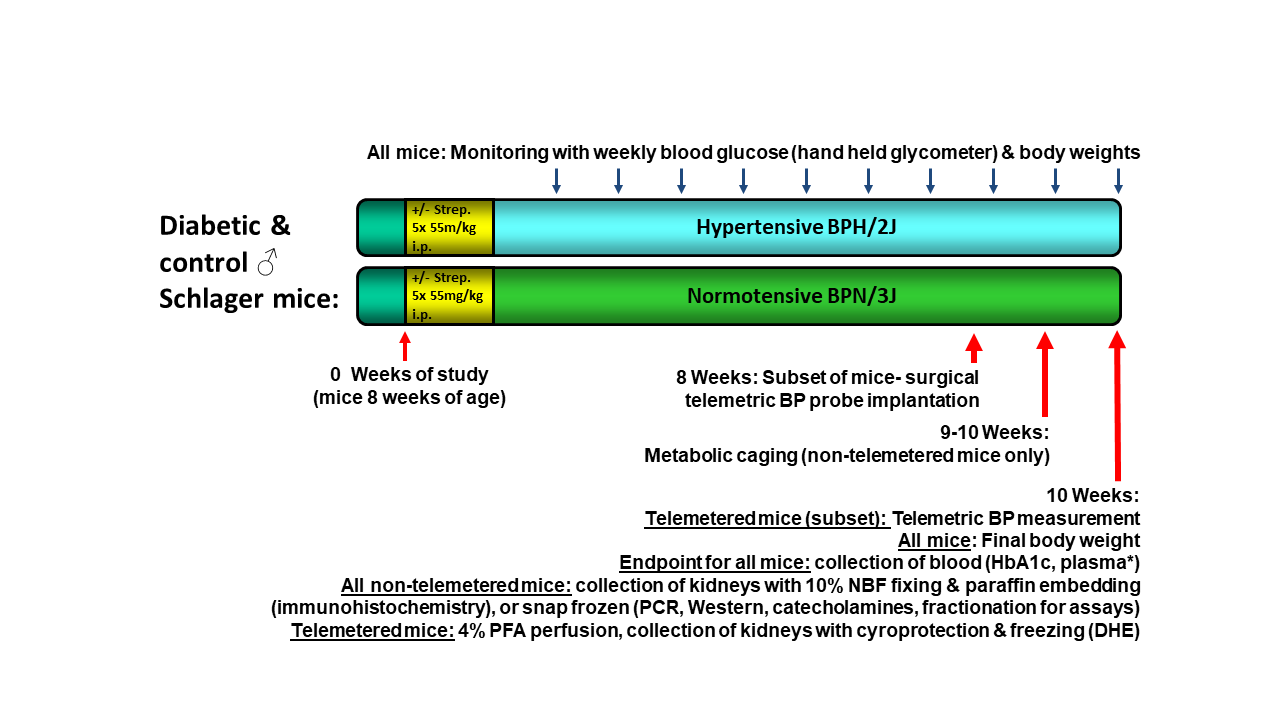


**Supplementary Figure 1.** Timelines for protocols and measurements. DHE: dihydroethidium staining; NBF: neutral buffered formalin; PFA: paraformaldehyde; Strep.: Streptozotocin. *Note that blood from all animals was used for HbA1c, plasma glucose and plasma lipid measurements (Table 1); perfusion of telemetered mice with 4% PFA precluded use of tissue for measures other than DHE staining.


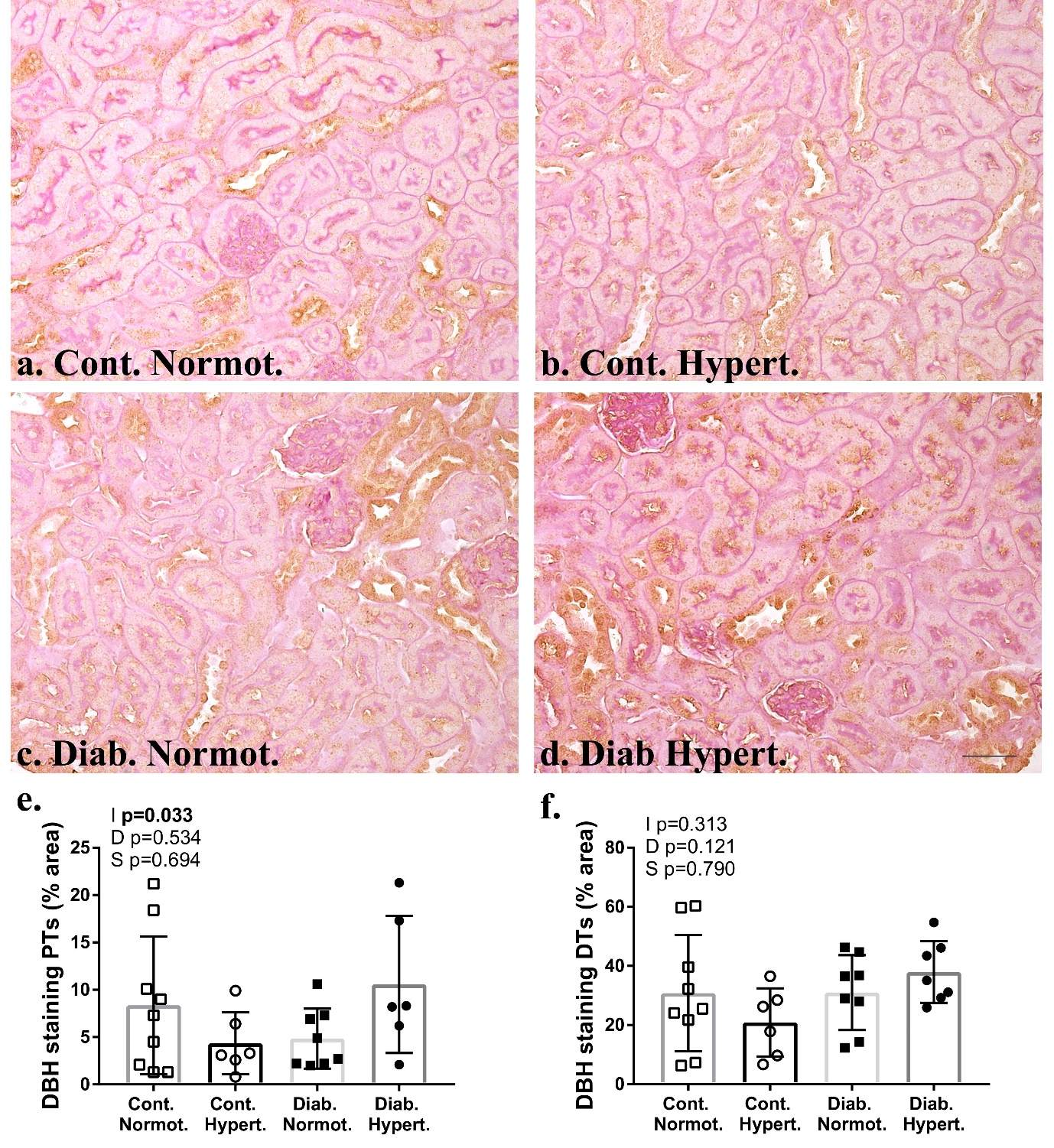


**Supplementary Figure 2.** Immunohistochemical localization in the renal cortex for dopamine β hydroxylase (DBH; **a**, **b**, **c**, **d**) (brown stain) with periodic acid Schiff used as a counterstain to clearly differentiate proximal (PTs) and distal tubules (DTs) for quantification (**e**, **f**). No significant differences in distribution of staining for DBH were found between groups. Overall there was a higher percentage area of DBH staining in distal as compared to proximal tubules (note difference in scale). 2-way ANOVA p-values: I=interaction, D=relative to diabetic status, S=relative to strain. Cont.- non-diabetic control; Diab.- diabetic; Normot.- normotensive BPN/3J mice; Hypert.- hypertensive BPH/2J mice. Scale bar: 50 µm.


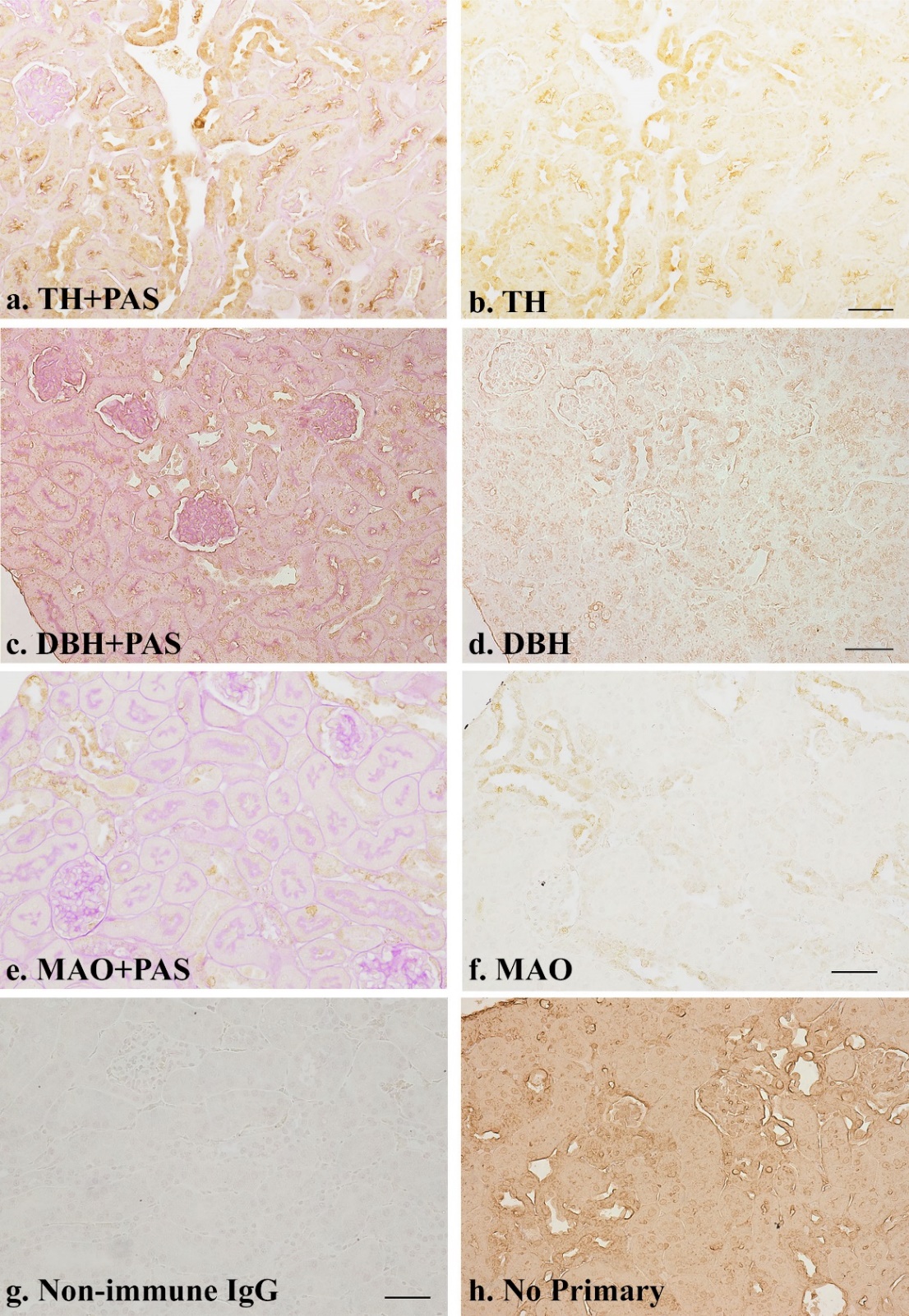


**Supplementary Figure 3.** Sections with and without periodic acid Schiff's (PAS) counterstaining and diaminobenzoate (DAB) immunostaining for tyrosine hydroxylase (TH, **a, b**) dopamine beta hydroxylase (DBH. c**, d**), and monoaminoxidase A (MAO, **e, f**) with (**a, c,** e) and without (**b, d, f**) periodic acid Schiff (PAS) staining. Use of PAS as a counterstain did not change the intensity of the DAB staining. Use of a non-immune primary IgG (Santa Cruz Biotechnology, Biolab, Clayton, Vic, Australia; followed by anti-Rabbit (Vector Laboratories) secondary, as per methods) did not result in staining (**g**) and incubation with neat rabbit serum rather than primary antibody resulted in no specific staining (**h**). Scale bars: 50 µm.


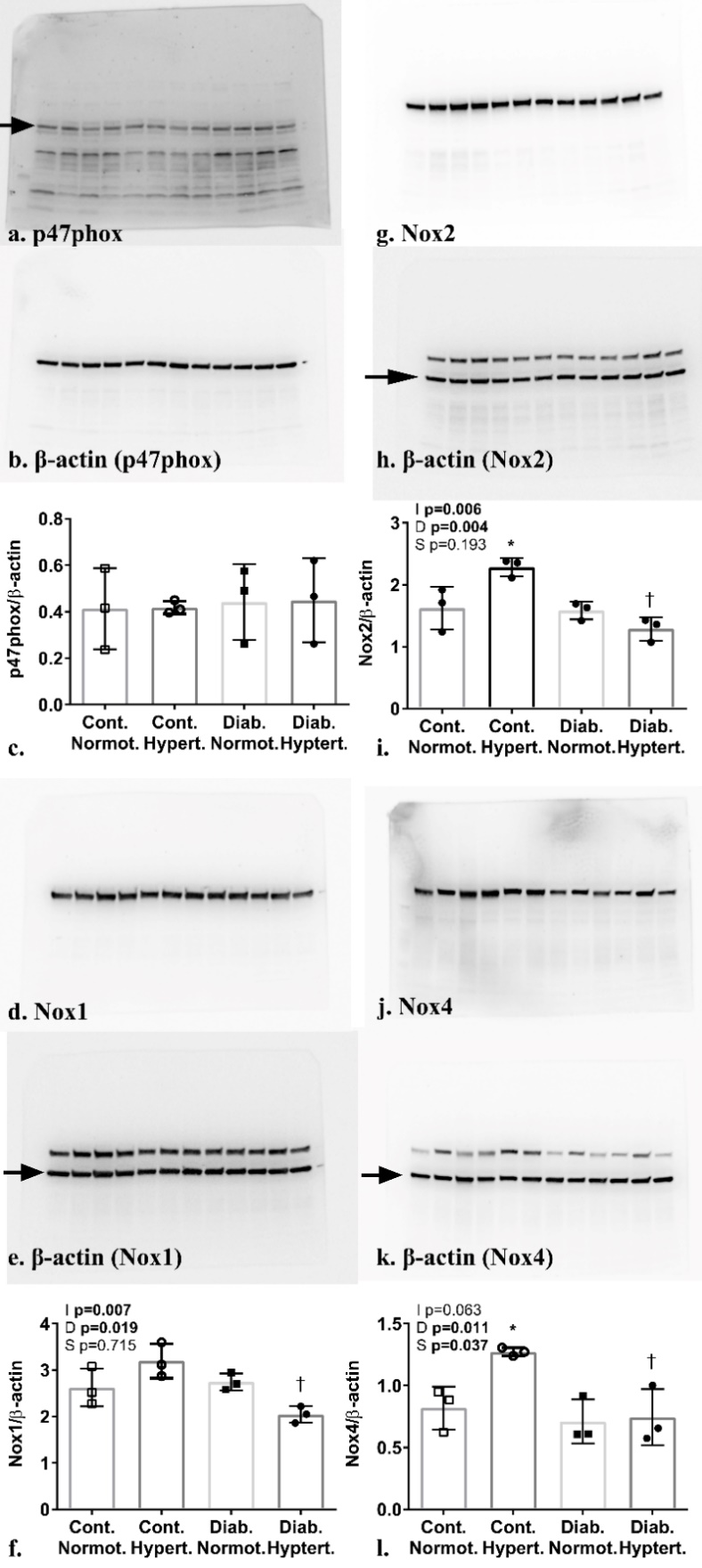


**Supplementary Figure 4**. Western blots: p47phox (**a, b, c**), Nox1 (**d, e, f**), Nox2 (**g, h, i**) and Nox4 (**j, k, l**) and their corresponding loading controls (β-actin) in renal cortical samples. Cont.- non-diabetic control; Diab.- diabetic; Normot.- normotensive BPN/3J mice; Hypert.- hypertensive BPH/2J mice. Antibodies: p47phox (rabbit polyclonal, Sigma), Nox1 (rabbit polyclonal, ProSci Inc., Poway, CA, USA), Nox2 (rabbit monoclonal, Abcam), Nox4 (Rabbit polyclonal, Abcam) run as per methods in main manuscript. Arrows indicate bands of the correct size. Note incomplete stripping of the membrane in e., h. & k. n=3/gp. 2-way ANOVA p-values: I=interaction, D=relative to diabetic status, S=relative to strain. t-test p<0.05: * vs Cont. Normot.; † vs Cont. Hypert.


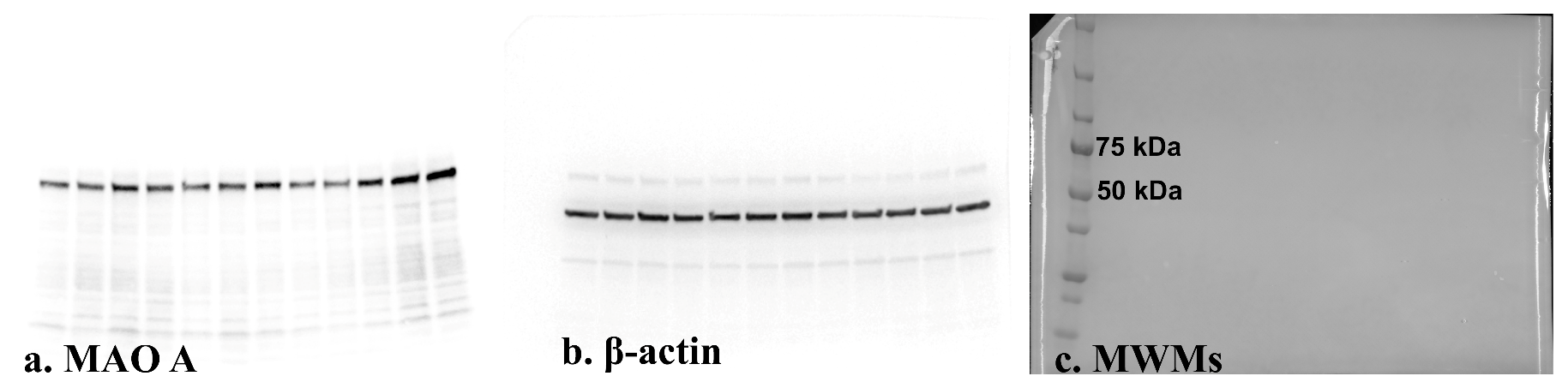


**Supplementary Figure 5**. Original images for Western blots used in figure 4a. Blots show renal cortical samples (n=3 animals/group) for MAO A (**a.**), β-actin (**b.**) and molecular weight markers (MWMs) (**c.;** Precision Plus protein standards, Bio-Rad).

**Supplementary Table 1.** Gene expression for markers of fibrosis and components of the renin-angiotensin system from renal cortex of mice. n=4-9. Data represent mean ± SEM. p>0.05: * vs Cont. Hypert.; † vs Diab Hypert.; ‡ vs Diab. Normot.

| Gene | Cont. Normot. | Cont. Hypert. | Diab. Normot. | Diab. Hypert. |
| --- | --- | --- | --- | --- |
| **Fibrotic** |  |  |  |  |
| *tgfb1* | 1.0 ± 0.1 | 1.2 ± 0.4 | 0.9 ± 0.3 | 1.1 ± 0.2 |
| *ednra* (ETA receptor) | 1.0 ± 0.2 | 1.7 ± 0.4 | 1.3 ± 0.5 | 2.6 ± 0.7 |
| *ednrb* (ETB receptor) | 1.0 ± 0.2 | 1.3 ± 0.1 | 1.9 ± 0.8 | 2.0 ± 0.5 |
| **Renin-angiotensin System** |  |  |  |  |
| *ace2* | 1.0 ± 0.1 | 1.4 ± 0.2 | 1.2 ± 0.2 | 1.7 ± 0.3 |
| *mas1* (mas receptor) | 1.0 ± 0.2 | 0.8 ± 0.2 | 1.9 ± 0.8 | 0.9 ± 0.2 |

**Supplementary Table 2** PCR primer sets and probes for genes listed in table 1 with encoded proteins in brackets.

| Current Gene Nomenclature | Probe Sequence  5’FAM-3’TAMRA | Forward Primer 5’-3’ | Reverse Primer 5’-3’ | GeneBank Accession Number |
| --- | --- | --- | --- | --- |
| *dbh* | 6-FAM CTACGCCCCTATCTC | GGGATATGCTCAAGGCTCTGTATG | AGCAGAAGTCTTGTTGCAATG | NM_138942 |
| *adra1b*  (α adrenoceptor 1b subunit) | SYBR | GCTACTTCAACAGCTGCCTCAAC | AGGCGCGTTTGAACTCCT | NM_001284380 |
| *adrb1*  (β1 adrenoceptor) | SYBR | CCGAAAGCAGGTGAATGCA | GCTTTCTGTACGTCTCGGATGA | NM_007419 |
| *slc6a2*  (norepinephrine transporter) | SYBR | CCTGGTGGCCCAGAGAGA | CACATTGCCAGGTTCAGATAGC | NM_009209 |
| *maoa* | SYBR | ACCAAGCCAGATGGGTCACT | CAGCTTTCCGGGCAAGAA | NM_173740 |
| *maob* | SYBR | GGGAAGATTCCAGAGGATGAAA | GTGCAGGGACATCCACAGACT | NM_172778 |
| *comt* | SYBR | AGGAGTGGGCCATGAACGT | CCGAATCACTGCATCCATGA | NM_001111062 |
| *ren1* (renin) | 6- FAM TTCGCCTTGGCCCGC | GGCATAACAATCGCATTGGA | GGACAAAGCCAGACAAAATGG | NM_031192 |
| *ace* | 6- FAM CCACCTGCTGGTCC | GGTCTGCTTCCCCAACAAGA | TCGTGAGGAAGCCAGGATGT | J03940 |
| *ace2* | 6- FAM TTGTCTGCCACCCCACA | GGGCAAACTCTATGCTGACTGA | TGTGACCTTTGTACACATCTTGATTC | AB053181 |
| *agtr1*  (AT1A receptor) | 6-FAM TCTCGCCTCCGCCG | CCATTGTCCACCCGATGAA | TGACTTTGGCCACCAGCAT | NM_177322 |
| *agtr2*  (AT2 receptor) | 6-FAM AATCGGTCATCTACCC | ATTACCTGCATGAGTGTCGATAGG | GGGATTCCTTCTTTGAGACAGAAA | BC003811 |
| *mas1*  (mas receptor) | 6- FAM ACCACCATGGAGTATGT | CCCAAGCACCAGTCAGCATT | CTCTTCTCCGCTGTCAATACACA | NM_008552 |
| *nox1* | 6- FAM CTAGAATAGCTACTGCCCACC | GACCAATGTGGGACAATGAGTTT | CCCCCACCGCAGACTTG | NM_172203 |
| *cybb* (nox2/gp91phox) | 6- FAM CAACTGGACAGGAACCT | AGTGCGTGTTGCTCGACAAG | CCAAGCTACCATCTTATGGAAAGTG | U43384 |
| *nox4* | 6- FAM CATTTTGCTATTTCATCAAA | AAAAATATCACACACTGAATTCGAGACT | TGGGTCCACAGCAGAAAACTC | NM_015760 |
| *ncf1* (p47phox) | 6- FAM CCCAGCCTTCTGCAGAT | CCGGCTATTTCCCATCCAT | TCGCTGGGCCTGGGTTAT | NM_010876 |
| *sod1* (CuZnSOD) | 6- FAM TGTGATCTCACTCTCAGGAG | GGACGGTGTGGCCAATGT | CGGCCAATGATGGAATGC | XM_128337 |
| *sod2* (MnSOD) | 6- FAM CCTGAGCCCTAAGGG | GGGACATATTAATCACACCATTTTCTG | CCCAAAGTCACGCTTGATAGC | X04972 |
| *cat* (catalase) | 6-FAM CACTGACGTCCACCC | TTCAGAAGAAAGCGGTCAAGAAT | GATGCGGGCCCCATAGTC | NM_009804 |
| *acta2*  (α2 smooth muscle actin) | 6- FAM TGCCAGATCTTTTCC | GACGCTGAAGTATCCGATAGAACA | GGCCACACGAAGCTCGTTAT | NM_007392 |
| *fn1*  (fibronectin) | 6- FAM CCCCGTCAGGCTTA | ACATGGCTTTAGGCGGACAA | ACATTCGGCAGGTATGGTCTTG | M10905 |
| *col4a1*  (collagen IV) | 6- FAM CAGTGCCCTAACGGT | GGCGGTACACAGTCAGACCAT | GGAATAGCCGATCCACAGTGA | J04694 |
| *tgfb1* | 6- FAM AAAGCCCTGTATTCCGT | GCAGTGGCTGAACCAAGGA | GCAGTGAGCGCTGAATCGA | NM_011577 |
| *ctgf* | 6- FAM ACTGCCTGGTCCAGAC | GCTGCCTACCGACTGGAAGA | CTTAGAACAGGCGCTCCACTCT | BC006783 |
| *vegfa* (VEGF) | 6- FAM CTGTACCTCCACCATGC | GCACTGGACCCTGGCTTTACT | ATGGGACTTCTGCTCTCCTTCTG | M95200 |
| *ednra*  (ETA receptor) | 6-FAM TGTGCCTTCAAGTCC | AACTAACCCTCCGCAGAAACAC | GCGTAATGGTTGTTTCCATCCT | NM_010332 |
| *ednrb*  (ETB receptor) | 6-FAM CTGTCCCCGAAGCC | TGGGTGGTCTCTGTGGTTCTG | GTCCGACGTAATCATATCAAAACCT | NM_007904 |
| *rela*  (p65 subunit of NFκB) | 6-FAM AGCTCAAGATCTGCCG | TCTCACATCCGATTTTTGATAACC | CGAGGCAGCTCCCAGAGTT | M61909 |

**Supplementary Table 3** A summary of the significant differences between diabetic and non-diabetic control, normotensive and hypertensive and also diabetic normotensive and diabetic hypertensive Schlager mice. ↓: significantly lower parameter of the former versus the latter group. ↑: significantly greater parameter of the former versus the latter group; ^a^ normotensive animals only; ^b^ hypertensive mice only; ^2-way ANOVA. BW- body weight.

| **Parameter** | **Diabetic vs. Non-Diabetic Control** | **Hypertensive vs. Normotensive (Non-Diabetic)** | **Diabetic Hypertensive vs. Diabetic Normotensive** |
| --- | --- | --- | --- |
| **General Parameters** | ↓ BW;  ↑ water intake; ↑urine output;  ↑ Food Consumption;  ↑ Left & Right Kidney Weight/BW | ↑ BP; ↓ BW; ↑ Food Consumption^ | ↑ BP; ↓ BW |
| **Blood & Plasma** | ↑ plasma cholesterol; ↑ plasma glucose; ↑ HbA1c | - | ↑ HbA1c |
| **Renal Cortical Gene expression** | ↓*ace*^b^; ↑*adrab1*^  ↑ *slc6a* ^a^; ↑*nox1*^  ↑ *fn1*^b^; ↑ *maoa* ^b^; ↑ *ncf1* ^b^ | ↑ *agtr2*; ↑ *ace;*  ↑ *nox4*; ↑ *cat* | ↑ *agtr2*; ↑ *cat*; |
| **Renal Function** | ↑ albuminuria;  ↑ mesangial expansion;  ↑ urinary KIM-1^; ↓ plasma cystatin C | - | ↑ albuminuria |
| **Catecholaminergic**  **(Renal Cortex)** | ↓ Proximal Tubular TH Staining ^b^;  ↓ Dopamine^; ↓ Norepinephrine^ | ↑ Dopamine; ↑ Norepinephrine | ↑ Dopamine; ↑ Norepinephrine;  ↑ Tubular MAOA |
| **Oxidant and Anti-Oxidant** | ↑ DHE fluorescence | ↑ Mitochondrial Hydrogen Peroxide;  ↑ Cytosolic Catalase Activity | ↑ Mitochondrial Hydrogen Peroxide;  ↓ Cytosolic Catalase Activity |
